# Notes and News

**Published:** 1903-10-03

**Authors:** 


### Notes and News.

Dr. Louis Parkes, medical officer of Chelsea, has been appointed Consulting Sanitary Adviser to his Majesty's Works and Public Buildings. Dr. Louis Parkes succeeds the late Professor Corfield.

The existence of Chinatown, in San Francisco, is threatened. It is believed that it is the cause of the spread of the plague, and it is felt that the latter cannot be wholly stamped out unless Chinatown is abolished. Probably no one but a few curious visitors will regret such a decision.

It is reported that Metz, suffering recently from an epidemic of typhoid, received a message from the Emperor desiring that the strongest measures should be taken to overcome the disease. The message was placarded in the city with the desired effect, though not without arousing much indignation amongst the town officials.

A well thought-out scheme to arrest tuberculosis has been established in Belgium by an Anti-tuberculosis League. Through its agency dispensaries are being established which embrace a system of domiciliary visits to the patients, and as far as possible outdoor treatment. This latter can only be secured as yet to a very limited extent, as the League has only one sanatorium. With the influx of funds this will of course be rectified.

We have more than once urged the advisability of municipal control, or at any rate of rigorous inspection of the public milk supply, and we are glad therefore to be able to state that the Lambeth Borough Council, following Battersea's example, has decided to open a depôt for the sale of milk. It will be situated in the neighbourhood of three important hospitals, and we understand that the Council's decision is the outcome of representations on the part of the medical staff of one of these—the Royal Waterloo Hospital—who have pledged themselves to do all in their power to induce their patients to avail themselves of it.

There are now only eight cases of small-pox in Cambridge under treatment, and no fresh cases have been notified.

The Autumn Session of the West London Post-Graduate College commences on October 13th, when Sir William Church will deliver an address on Medical Education and Post-Graduate Study.

The new wing of the Mount Vernon Hospital for Consumption is to be opened by the Marquis of Zetland, President of the hospital, on October 27th. Already the exterior of the building is practically complete and has a most imposing appearance, and the fine site is now made the most of.

A contaminated water supply within the borough of Chelmsford caused 1,400 cases of diarrhœa, of which 14 were fatal. During the severe storms of this summer some manurial matter was washed from a garden into a service reservoir which was not bricked up above the surface of the ground.

Pei-tang is in a very unfortunate position at the present time. Both plague and cholera are raging, and 2,000 deaths are reported during the last two months. Pei-tang has only a population of 15,000. A sanitary commission has been appointed to prevent the spread of the diseases, and so far the larger towns in the vicinity of Pei-tang have not been contaminated.

The recent report of the Liverpool medical officer for health contains another strong testimony in favour of prompt re-vaccination in times of epidemic more especially. The report shows the favourable results of this very markedly. The medical officer is strongly in favour of the transference of the power to undertake free vaccination from the poor-law authorities to the Health Department in order to avoid delay now inevitable.

The owners of gymnasia evidently anticipate an era of prosperity stimulated by the attention which is now being paid to physical development. Without wishing to depreciate the use of a gymnasium as an adjunct to physical development, it will be an unfortunate day if the growing youths in our cities look to the gymnasium, and more especially to covered and walled-in gymnasia, as the best and surest means of improving their physique. If those eager to forward an improvement in the physical condition of the town dweller will devote their charity and energy to the encouragement and establishment of athletic clubs in the open, where fresh air, exercise, and recreation can be combined, a really valuable work will be done. It is true that two afternoons in the week would be the maximum amount of time available in the winter for this purpose, namely, Saturday and Sunday, but those two afternoons would be infinitely more valuable than three times the number of evenings spent in a less desirable atmosphere. The advocates of "exercise" are apt to overlook the fact that to merely develop the muscles at the expense of fatigue is not to build up the general health of the individual. The pale city dweller needs oxygen infinitely more than mere muscular exercise.

